# Supplementary material for: Online legal driving behavior monitoring for self-driving vehicles
Source: Nat Commun. 2024 Jan 9;15:408. doi: 10.1038/s41467-024-44694-5 (PMC10776857; doi:10.1038/s41467-024-44694-5)
Supplement: Supplementary file 1 — Supplementary Information [file 41467_2024_44694_MOESM1_ESM.pdf]

Supplementary Information

Title

Online Legal Driving Behavior Monitoring for Self-driving Vehicles

Authors

Wenhao Yu[1, †], Chengxiang Zhao[2, †], Hong Wang[1, ✉] , Jiaxin Liu[1], Xiaohan Ma[2], Yingkai Yang[1], Jun Li[1], Weida Wang[2], Xiaosong Hu[3, ✉], Ding Zhao[4]

† These authors contributed equally to this work.

Affiliations

[1] School of Vehicle and Mobility, Tsinghua University, 100084, Beijing, China

[2] School of Mechanical Engineering, Beijing Institute of Technology, 100084, Beijing, China

[3] Department of Mechanical and Vehicle Engineering, Chongqing University, 400044, Chongqing, China

[4] Department of Mechanical Engineering, Carnegie Mellon University, Pittsburgh, 15213, PA, USA

✉ Corresponding authors: hong_wang@tsinghua.edu.cn, xiaosonghu@ieee.org

This PDF file includes:

Supplementary Text, Figures, and Tables

Contents

1 Supplementary Information

1.1 Concepts involved in atomic propositions

1.2 Offline monitoring and online monitoring

2 Supplementary Method

2.1 Process of traffic laws formalization

2.2 Data filter principle for compliance threshold analysis

2.3 Calculation of TTI difference for online monitoring

3 Supplementary Result

3.1 Complexity analysis of subdivided articles

3.2 Results in the process of threshold analysis

3.3 Some interesting findings

1 Supplementary Information

1.1 Concepts involved in atomic propositions

Supplementary Table 1 provides concepts involved in atomic propositions mentioned in the main paper and the Supplementary Method.

Table 1 Concepts involved in atomic propositions

Label	Meaning
Object	
Ego	The ego vehicle
Tgt	The target traffic participant in specific scenarios
$\mathrm{Tgt_f}$	The Tgt closest to the ego vehicle in the Front(Ego) region
$\mathrm{Tgt_{fl}}$,$\mathrm{Tgt_{fr}}$,$\mathrm{Tgt_r}$, $\mathrm{Tgt_{rl}}$,$\mathrm{Tgt_{rr}}$	Similar to $\mathrm{Tgt_f}$, represent the Tgt closest to the ego vehicle in the corresponding region: FrontLeft(Ego), FrontRight(Ego), Rear(Ego), RearLeft(Ego), RearRight(Ego).
RVTL	The rear vehicle in the target lane when ego vehicle makes lane-change
$\mathrm{Tgt_{os}}$	The oncoming straight-moving vehicles when the ego crosses the intersection
$\mathrm{y}(i)$	Cubic fitting curve of ith lane line
N_{ml}	The number of mainlines in the same direction
WZ	The acceptable lateral wandering zone for lane-keeping vehicles
StopLine	The stop line of an intersection
IntersectionArea	The region formed by extending all StopLine in an intersection
VirtualLane_O	The virtual lane of oncoming straight-moving traffic when the ego crosses the intersection
Property	
x(obj)	The state value of obj, where the state $\mathrm{x} = [X, Y, \theta, \mathrm{vx}, \mathrm{vy}, \mathrm{ax}, \mathrm{ay}]$ represents longitudinal coordinate X, lateral coordinate Y, heading angle θ, longitudinal velocity vx, lateral velocity vy, longitudinal acceleration ax and lateral acceleration ay, respectively
RT(obj)	The type of lane that obj is currently in $\mathrm{RT} = \{M, R, A, D, E\}$, representing mainline, ramp, acceleration lane, deceleration lane and emergency lane, respectively
$\mathrm{L}(\mathrm{obj}, t)$	The lane ID to which obj belongs at time t
Area(obj)	The planar area occupied by the bounding box of obj
Front(obj)	The area in front of obj and located in the same lane
FrontLeft(obj)	The area in front of obj and located in left adjacent lane
FrontRight(obj)	The area in front of obj and located in right adjacent lane
Rear(obj)	The area behind of obj and located in the same lane
RearLeft(obj)	The area behind of obj and located in left adjacent lane
RearRight(obj)	The area behind of obj and located in right adjacent lane
TL(obj)	The current traffic light state that obj should obey. $\mathrm{TL} = \{R, G, Y\}$ represent red, green, and yellow lights, respectively
w(obj)	The width of obj
l(obj)	The length of obj
VehicleID(obj)	The vehicle ID recorded in dataset
Relationship	
SpdSignArea(obj)	The obj located in the speed sign management area
$\mathrm{overlap}(r_1, r_2)$	There is an overlap between regions r_1 and r_2
Calculate	
$\mathrm{dis}(\mathrm{obj}_1, \mathrm{obj}_2)$	Calculate the longitudinal distance at time t between obj_1 and obj_2 along the lane direction. obj_2 is in front of obj_1. $\mathrm{dis}(\mathrm{obj}_1, \mathrm{obj}_2) = \int_{X(\mathrm{obj}_1)}^{X(\mathrm{obj}_2)} \sqrt{1 + (\mathrm{y}(\mathrm{L}(\mathrm{obj}_1, t)))^2} dX - (\mathrm{l}(\mathrm{obj}_1) + \mathrm{l}(\mathrm{obj}_2))/2$
$\mathrm{TTCX}(\mathrm{obj}_1, \mathrm{obj}_2)$	Calculate the longitudinal TTC at time t between obj_1 and obj_2 along the lane direction. obj_2 is in front of obj_1. $\mathrm{TTCX}(\mathrm{obj}_1, \mathrm{obj}_2) = \mathrm{dis}(\mathrm{obj}_1, \mathrm{obj}_2)/(\mathrm{vx}(\mathrm{obj}_1) - \mathrm{vx}(\mathrm{obj}_1))$
$\mathrm{TTIdiff}(\mathrm{obj}_1, \mathrm{obj}_2)$	Calculate the time difference to intersection point of obj_1 and obj_2. $\mathrm{TTIdiff}(\mathrm{obj}_1, \mathrm{obj}_2) = \mathrm{TTI}(\mathrm{obj}_2) - \mathrm{TTI}(\mathrm{obj}_1)$

1.2 Offline monitoring and online monitoring

Offline monitoring is a law violation monitoring that judges the compliance of the driving behaviors of one or more vehicles in the whole scenario by obtaining the whole-period vehicles' behavior information. In contrast, online monitoring is a law violation monitoring that judges the compliance of driving behaviors of the ego vehicle or all vehicles from the start of the monitoring process to the current time using the observed or collected data. It should be noted that different purposes of online monitoring show certain differences. Among online monitoring, three kinds of purposes are proposed: 1) fact-based monitoring, 2) decision-based monitoring, and 3) prediction-based monitoring. The choice between online monitoring and offline monitoring will result in different formalization procedures and result formulas. Each kind of purpose is comprised in detail as Supplementary Table 2.

Table 2 The comparisons of different monitoring

Comparison	Offline monitoring	Online monitoring		
		Fact-based law violation monitoring	Decision-based law violation monitoring	Prediction-based law violation monitoring
Application	1). Used for offline evaluation of SV tasks of a vehicle; 2).Used in the roadside equipment to provide third-party monitoring for vehicle behaviors	1). Used at an SV for online monitor of SV's behaviors and division of accident responsibility; 2). Used in the roadside equipment for online third-party monitor of vehicle behavior violations	Used in a vehicle for online management of autonomous driving behaviors and online interference response for illegal decision-making	Used in a vehicle for online management of autonomous driving behaviors and online interference response for possible violations
Information	Ego vehicle: all time periods of data<br>Other vehicle: all time periods of data	Ego vehicle: current and past periods of data<br>Other vehicle: current and past periods of collected data	Ego vehicle: current and past periods of data and decision-making data<br>Other vehicle: current and past periods of collected data	Ego vehicle: current and past periods of data and decision-making data<br>Other vehicle: current and past periods of collected data and the corresponding future prediction data
Overall judgment difficulty	Easy	Hard	Medium	Medium
Results	Unchangeable	Unchangeable	Varying with decisions	Varying with decisions, control outputs and others' behaviors
Decision friendly	None	Bad	Good	Best

In Supplementary Table 2, online monitoring is divided into three types according to the information used for monitoring. The fact-based monitoring uses historical and current vehicle behavior data. In this type of monitoring, once a law violation behavior is found, the violation result is an established fact that cannot be changed. Therefore, this type of monitoring can be used on both the vehicle side and the road equipment side for recording violation behaviors of the vehicles, and the result can be used as a reference for accident responsibility division. Besides historical and current vehicle behavior data, when the ego vehicle's decision data are involved, online monitoring is given the ability to "foresee" the future actions of the ego vehicle. This type of monitoring is regarded as decision-based law violation monitoring, and it can be used only for ego vehicles. However, this monitoring type can tell whether the ego vehicle will break the traffic law if following the current decision. Also, the decision-making system can read the monitor's output to adjust its decision to comply with the traffic law. Therefore, the monitoring result changes with the decision of the ego vehicle. Furthermore, the traffic law restrains the relationships between traffic participants. Thus, if it is required that the monitoring result has the best law compliance guiding significance, the prediction behaviors of other participants should also be considered, and this represents the prediction-based law violation monitoring. This monitoring type combines historical and current vehicle behavior data with the ego vehicle's decision and perception data to judge the law compliance of the decision in the prediction range. Owing to its heavy reliance on prediction, the monitoring result is unstable and varies with the decisions, other participants' behaviors and their predictions. However, this monitoring type is the most decision-friendly monitoring, and its result gives the best advance quantity to adjust the decision.

If the monitored vehicle is a white box for offline monitoring or only in certain given scenarios, it will be easy to select law monitoring algorithms to determine behavior violations for vehicle decisions or scenario types. However, when facing a black box vehicle and in a free-run situation, performing online monitoring is relatively challenging because it is necessary to estimate a vehicle' next action on the whole trajectory and which law is convenient for a particular case. Therefore, without the whole-trajectory data and using only past and current data that the ego vehicle collected, it is difficult to monitor behavior violations for the fact-based monitor because it is required to set more judgment conditions to determine which law is suitable for the current scenario. Furthermore, right-of-way monitoring is even more challenging because other traffic participants are involved. These participants' behaviors can lead to a situation where much more judgment conditions need to be discussed, and more thresholds should be considered. By using the whole-trajectory data of all participants or the ego vehicle's decision and prediction data, the monitor task will become easier to perform because the future data can reduce the condition classification discussions. This is the main reason the fact-based monitor is the most challenging to achieve.

2 Supplementary Method

2.1 Process of traffic laws formalization

Countries and regions around the world have their own distinct road traffic laws shaped by local cultures, histories, and social backgrounds. Although there are variations in laws among countries, these differences primarily lie in the thresholds of constraints on different behaviors. The behaviors constrained by the traffic laws and the meanings of the constraints are generally consistent. Therefore, for any subdivided article, we first extract semantic elements based

on behavior, scene, participant, environment and threshold. The scene represents the traffic elements mentioned in the regulations, such as signs, markings, and location areas. Participants refer to the traffic participants involved in the regulations, and the environment represents additional environmental factors such as weather and lighting. These three elements together form the operational environment (OE) of the article. Behavior refers to the maneuvers that the vehicle should or should not take under this OE. The threshold limits the permissible boundaries of the behavior margin, and some of these thresholds are ambiguous and need to be further determined through behavior analysis, data mining, or other means. The standardized formalized process is shown in Supplementary Fig.1.

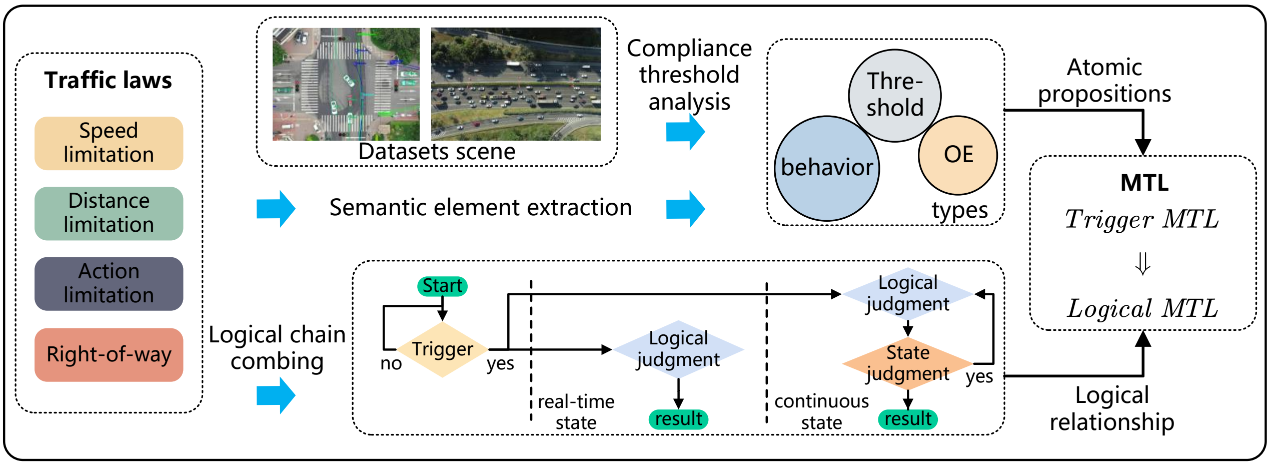

Fig. 1 Standardized formalized process of traffic laws. Each article will extract different types of semantic elements, which will then be transformed into calculable atomic propositions. Simultaneously, the temporal logic relationships of the article will be obtained through logical chain sorting, and combined with atomic propositions to form MTL expression.

Some semantic elements can be directly translated into state variables, e.g., vehicle speed, and current lane. Other semantic elements must be further abstracted into computable functions, e.g., "not impede the normal running of rear vehicles in the relevant lanes" in Article 44 is abstracted as $\mathrm{dis}(\mathrm{Ego}, \mathrm{RVTL})$ exceeding a certain threshold. All elements should be obtained or calculated. The abstracted computable functions, combined with thresholds, are used to construct state judgment expressions, thereby generating the construction of computable atomic propositions. Notably, different application purposes after formalization can affect the construction of computable atomic propositions. To ensure computability, the clarification of the obtainable inputs is required in advance.

Before constructing MTL expressions, the logical chain of the subdivided article must be combed. First, the trigger conditions should be clarified to ensure SVs consider articles only relevant to the current scenario. After the trigger conditions are satisfied, we must identify logical definitions that need to be satisfied and the temporal logical relationships between them. Logic states can be broadly classified into two types: real-time state and continuous state. Real-time state characterizes whether the current behavior of the vehicle is compliant with the traffic law, e.g., whether the current following distance is too close. While continuous state characterizes whether the behavior of the vehicle is illegal over a period, e.g., the judgment of interval average speed limitation.

Until now, the MTL formula can be constructed with the abstracted computable atomic propositions and the combed logical chain. Thus, each natural language article is formalized into a standardized mathematical description that includes input requirements, trigger conditions, logical definitions and judgment thresholds.

2.2 Data filter principle for compliance threshold analysis

In this section, we provide more details on the data filter principles for the compliance threshold analysis. The thresholds for articles related to highway were analyzed using the AD4CHE dataset. Each segment of the dataset has an approximate length of 140 m. Therefore, there exist many incomplete lane change maneuvers (LCM). The thresholds of $T_{\mathrm{cl\_min}}$, $\mathrm{TTC}_{\mathrm{cl\_min}}$ and $d_{\mathrm{cl\_min}}$ require complete LCM. To filter the complete LCM, the behavior of a vehicle should adhere to the following logic:

Assume that the initial and final frame of the ego vehicle are f_0 and f_{end}, respectively.

$$\begin{aligned}
&G_{[f_0]}(\neg\mathrm{overlap}(\mathrm{Area}(\mathrm{Ego}), \mathrm{y}(\mathrm{L}(\mathrm{Ego}, f_0))|\mathrm{y}(\mathrm{L}(\mathrm{Ego}, f_0) + 1)))\wedge \\
&G_{[f_{\mathrm{end}}]}(\neg\mathrm{overlap}(\mathrm{Area}(\mathrm{Ego}), \mathrm{y}(\mathrm{L}(\mathrm{Ego}, f_0))|\mathrm{y}(\mathrm{L}(\mathrm{Ego}, f_0) + 1))) \Rightarrow \\
&\exists[f_1, f_2] \in [f_0, f_{\mathrm{end}}]\wedge \\
&G_{[f_1, f_2]}(\mathrm{overlap}(\mathrm{Area}(\mathrm{Ego}), \mathrm{y}(\mathrm{L}(\mathrm{Ego}, f_0))|\mathrm{y}(\mathrm{L}(\mathrm{Ego}, f_0) + 1)) \wedge \mathrm{L}(\mathrm{Ego}, f_1) \neq \mathrm{L}(\mathrm{Ego}, f_2))
\end{aligned} \tag{1}$$

where f_1 and f_2 are the first and last frames that the vehicle overlaps with the lane line, respectively. $[f_1, f_2]$ is a subset of $[f_0, f_{\text{end}}]$, and $\text{L}(\text{Ego}, f)$ represents the Lane ID of ego vehicle at frame f.

1) To analyze $T_{\text{cl\_min}}$, after filtering all instances of complete LCM, further selection or calculations are required for these instances. For the maximum allowable time to drive on the lane line ($t_{\text{cl\_max}}$), the frame rate of the AD4CHE dataset is 30 Hz, the lane change time ($t_{\text{cl}} = (f_2 - f_1)/30$) is being counted for each vehicle.

2) To analyze $\text{TTC}_{\text{cl\_min}}$, when the trigger condition is first activated, beside Equation 1, the preceding vehicle must also maintain lane-keeping during the LCM. Only instances with a TTC in the 0-20 s range will be retained.

$$\begin{aligned} &G_{[f_1,f_2]}(\exists \text{Tgt}_{\text{f}} \wedge \neg \text{overlap}(\text{Area}(\text{Tgt}_{\text{f}}), \text{y}(\text{L}(\text{Ego}, f_0))|\text{y}(\text{L}(\text{Ego}, f_0) + 1))) \\ &\wedge G_{[f_1]}(0 < \text{TTCX}(\text{Ego}, \text{Tgt}_{\text{f}}) < 20) \end{aligned} \tag{2}$$

3) To analyze $d_{\text{cl\_min}}$, we mentioned four criteria used to filter instances with complete and reasonable behavior:
(1) The ego vehicle has a successful LCM: this criteria has been mentioned in equation 1.
(2) The RVTL maintains a constant vehicle ID throughout the scenario:

$$G_{[f_1,f_2]}(\text{VehicleID}(\text{RVTL}) = \text{VehicleID}(\text{RVTL}, f_1)) \tag{3}$$

(3) The RVTL is within the wandering zone when the trigger condition is first activated:

$$G_{[f_1]}(Y(\text{RVTL}) \in \text{WZ}) \tag{4}$$

(4) The RVTL acceleration is greater than -0.7 m/s^2 before the rigger condition activates:

$$G_{[f_{\text{RVTL0}}, f_1]}(\min(\text{ax}(\text{RVTL})) > -0.7) \tag{5}$$

Where VehicleID(RVTL) is the vehicle ID of RVTL recorded in dataset, and $\text{VehicleID}(\text{RVTL}, f)$ represents the vehicle ID of RVTL at frame f. WZ is the wandering zone mentioned in the main paper.

Two indicators are established for "No Changes" principle: the minimum acceleration of RVTL and its deviation from the centerline. When the RVTL exhibits the following behaviors, we consider it to be interfered:
(1) Significant deceleration:

$$G_{[f_{\text{RVTL0}}, f_1]}(\min(\text{ax}(\text{RVTL})) > -0.7) \wedge G_{[f_1,f_2]}(\min(\text{ax}(\text{RVTL})) < -0.7) \tag{6}$$

(2) Significant avoidance:

$$G_{[f_1]}(Y(\text{RVTL}) \in \text{WZ}) \wedge G_{[f_2]}(Y(\text{RVTL}) \notin \text{WZ}) \tag{7}$$

where f_{RVTL0} is the initial frame of RVTL.

At the intersection, the thresholds of minimum allowable time difference to the intersection point ($\text{TTI}_{\text{diff\_min}}$) were analyzed using the SIND dataset. For each set of left-turning and straight-moving conflict events, assume that the intersection point is P_{i}. The frame at which the left-turning vehicle arrives at P_{i} is f_{lP_i}. And the frame at which the straight-moving vehicle arrives at P_{i} is f_{sP_i}. Each set of instances was retained where the time interval between left-turning and straight-moving vehicles passing through the intersection point of the trajectories was less than 5 seconds ($|f_{\text{lP}_\text{i}} - f_{\text{sP}_\text{i}}|/10 < 5$, the frame rate of the SIND dataset is 10 Hz). With the retained instances, first of all, we extract the effective interval for each set of instances. Similarly, assume that the frame at which the left-turning vehicle starts overlapping with the stop line is f_{l0} and at which the straight-moving vehicle starts overlapping with the stop line is f_{s0}. The start frame of the effective interval $f_{\text{int},0}$ and the end frame $f_{\text{int,end}}$ are defined as:

$$f_{\text{int},0} = \max(f_{\text{s0}}, f_{\text{l0}}) \tag{8}$$

$$f_{\text{int,end}} = \min(f_{\text{lP}_\text{i}}, f_{\text{sP}_\text{i}}) \tag{9}$$

According to the position and velocity at each frame in effective interval, the sequence of TTI_{left} and TTI_{str} can be recorded. The calculation of TTI has been introduced in the main paper.

$$\text{TTI}_{\text{left}} = [\text{TTI}_{\text{left},f_{\text{int},0}}, \text{TTI}_{\text{left},f_{\text{int},0}+1}, ..., \text{TTI}_{\text{left},f_{\text{int,end}}}] \quad \text{TTI}_{\text{str}} = [\text{TTI}_{\text{str},f_{\text{int},0}}, \text{TTI}_{\text{str},f_{\text{int},0}+1}, ..., \text{TTI}_{\text{str},f_{\text{int,end}}}] \tag{10}$$

With the sequence of TTI_{left} and TTI_{str}, the conflicts between left-turning and straight-moving traffic are categorized into the following situations:

1) Straight-moving vehicle passes first

$$G_{[f_{\text{int},0}]}(\text{TTI}_{\text{left},f_{\text{int},0}} > \text{TTI}_{\text{str},f_{\text{int},0}}) \wedge G_{[f_{\text{int,end}}]}(\text{TTI}_{\text{left},f_{\text{int,end}}} > \text{TTI}_{\text{str},f_{\text{int,end}}}) \tag{11}$$

2) Left-turn vehicle gives way

$$G_{[f_{\rm int,0}]}(\mathrm{TTI}_{\mathrm{left},f_{\rm int,0}} < \mathrm{TTI}_{\mathrm{str},f_{\rm int,0}}) \wedge G_{[f_{\rm int,end}]}(\mathrm{TTI}_{\mathrm{left},f_{\rm int,end}} > \mathrm{TTI}_{\mathrm{str},f_{\rm int,end}}) \tag{12}$$

3) Left-turn vehicle passes first

$$G_{[f_{\rm int,0}]}(\mathrm{TTI}_{\mathrm{left},f_{\rm int,0}} < \mathrm{TTI}_{\mathrm{str},f_{\rm int,0}}) \wedge G_{[f_{\rm int,end}]}(\mathrm{TTI}_{\mathrm{left},f_{\rm int,end}} < \mathrm{TTI}_{\mathrm{str},f_{\rm int,end}}) \tag{13}$$

4) Left-turn vehicle rush

$$G_{[f_{\rm int,0}]}(\mathrm{TTI}_{\mathrm{left},f_{\rm int,0}} > \mathrm{TTI}_{\mathrm{str},f_{\rm int,0}}) \wedge G_{[f_{\rm int,end}]}(\mathrm{TTI}_{\mathrm{left},f_{\rm int,end}} < \mathrm{TTI}_{\mathrm{str},f_{\rm int,end}}) \tag{14}$$

After filtering and classifying, we retained 554 valid instances, comprising 82 instances of straight-moving vehicles passing first, 21 instances of left-turn vehicles giving way, 388 instances of left-turn vehicles passing first, and 63 instances of left-turn vehicles rushing.

The minimum acceleration of the straight-moving vehicle is used for “No Changes” principle. When the straight-moving vehicle exhibits the following behaviors, we consider it to be interfered:

$$G_{[f_{s0},f_{\rm int,1}]}(\min(\mathrm{ax}(\mathrm{Tgt}_{\rm os})) > -0.7) \wedge G_{[f_{\rm int,1},f_{\rm int,end}]}(\min(\mathrm{ax}(\mathrm{Tgt}_{\rm os})) < -0.7) \tag{15}$$

where $f_{\rm int,1}$ is the first frame that the left-turning vehicle intrudes into the virtual lane of oncoming straight-moving vehicle. Tgt_{os} is the oncoming straight-moving vehicles.

To determine the “No Crash” principle, the adaptive RSS distance $d_{\rm aRSS}$ of the straight-moving vehicle is introduced in the main paper. When the left-turning vehicle first intrudes into the virtual lane of the straight-moving vehicle, the distance of these two vehicles should greater than $d_{\rm aRSS}$:

$$G_{[f_{\rm int,1}]}(d_{\rm realstr} > d_{\rm aRSS}) \tag{16}$$

where $d_{\rm realstr}$ represents the distance to the intersection point $P_{\rm i}$ for the straight-moving vehicle.

2.3 Calculation of TTI difference for online monitoring

The constant turn rate and velocity model is utilized to calculate the intersection point of the ego vehicle’s trajectory with that of the corresponding straight-moving vehicle when the ego vehicle encroaches on the virtual lane line of the oncoming straight-moving traffic. The intersection points $(X_{\rm int}, Y_{\rm int})$ can be easily calculated using the intersection formula of line segments, while each sampling point of the obj’s trajectory can be calculated as follow:

$$\begin{cases} X_{k+1} = X_k + v(\mathrm{obj}) \cdot \cos\left(\theta(\mathrm{obj}) + \frac{\omega(\mathrm{obj})\cdot\Delta t}{2}\right) \cdot \Delta t \\ Y_{k+1} = Y_k + v(\mathrm{obj}) \cdot \sin\left(\theta(\mathrm{obj}) + \frac{\omega(\mathrm{obj})\cdot\Delta t}{2}\right) \cdot \Delta t \end{cases} \tag{17}$$

where, (X_k, Y_k) represents the $k^{\rm th}$ sample point in planning trajectory, $v(\mathrm{obj}) = \sqrt{(vx(\mathrm{obj})^2 + vy(\mathrm{obj})^2)}$ is the resultant velocity of obj, Δt is the sample time, $\omega(\mathrm{obj})$ is the heading angular velocity, and $\omega(\mathrm{obj}) = v(\mathrm{obj})/r$, in which, r is the radius of the centerline for the corresponding virtual lane.

When the ego vehicle turns left, with the intersection points $(X_{\rm int}, Y_{\rm int})$ and sequence of trajectory points, the TTI of the ego vehicle (TTI(Ego)) and oncoming straight-moving vehicle ($\mathrm{TTI}(\mathrm{Tgt}_{\rm os})$) can be calculated in real-time.

$$\mathrm{TTI}(\mathrm{obj}) = \frac{l_{\rm traj}}{v(\mathrm{obj})} \tag{18}$$

$$l_{\rm traj} = \left(\sum_{k=1}^{N-2} \sqrt{(X_{k+1} - X_k)^2 + (Y_{k+1} - Y_k)^2}\right) + \sqrt{(X_{\rm int} - X_{N-1})^2 + (Y_{\rm int} - Y_{N-1})^2} - \frac{l(\mathrm{obj})}{2} \tag{19}$$

where $l_{\rm traj}$ represents the length of the trajectory between the vehicle and the intersection point; N represents the minimum number of sampling points for trajectories that contain intersection point.

Then, the TTIdiff can be calculated as follow:

$$\mathrm{TTIdiff}(\mathrm{Ego}, \mathrm{Tgt}_{\rm os}) = \mathrm{TTI}(\mathrm{Tgt}_{\rm os}) - \mathrm{TTI}(\mathrm{Ego}) \tag{20}$$

3 Supplementary Result

3.1 Complexity analysis of subdivided articles

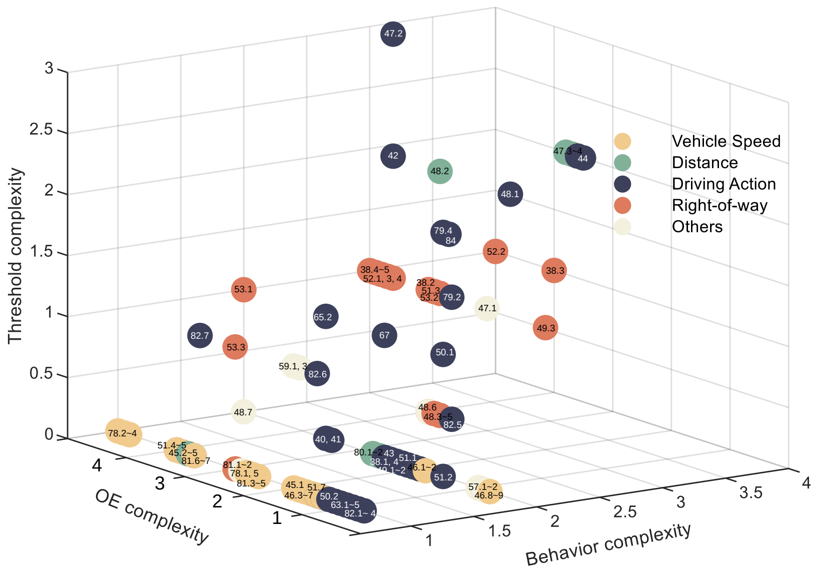

Fig. 2 Complexity Analysis of Subdivided articles. Each article will be assessed from three aspects: 1) OE complexity, 2) behavior complexity, and 3) threshold complexity, which represent the number of OE elements, the number of atomic propositions related to behavior judgments and the number of thresholds for ambiguous expressions respectively.

Based on different types of restrictions in traffic laws, we have subdivided 25 articles related to driving in The Regulation on the Implementation of the Law of the People's Republic of China on Road Traffic Safety into 93 detailed articles. In the formalization process, we evaluated and counted the formalization difficulty of each article. The difficulty of formalization primarily includes three aspects: 1) OE complexity, 2) behavior complexity, and 3) threshold complexity. The OE complexity is the number of OE elements included in the article. The OE elements include scene elements, participant elements, and environment elements. The more OE elements there are, the more complex the scenarios involved in the article. Behavior complexity refers to the number of atomic propositions related to behavior judgments involved in the article, which can represent the difficulty of judgment for this article. Threshold complexity refers to the number of thresholds for ambiguous expressions involved in an article. As shown in Supplementary Fig.2, all subdivided articles are evaluated and the number in each circle represents the entry of the article.

The overall complexity is the sum of the three complexities. The articles with an overall complexity greater than 5 are mainly related to driving actions and right-of-way restrictions. While articles about vehicle speed and other articles can be simply indicated by the scene and the thresholds do not contain interaction with other traffic participants, driving action and right-of-way restrictions mainly need to consider the interactions with other traffic participants and include behaviors that a single atomic proposition cannot simply express, such as "keep the necessary safety distance", and "slow down and drive to the right". The more interactions involved in an article, the higher the atomic proposition complexity will be. Moreover, ambiguous compliance thresholds need further analysis.

3.2 Results in the process of threshold analysis

When analyze "not impeding" the RVTL when making lane-change, we try to utilize the "No Changes" principle to analyze the deceleration of RVTL with the distance when the trigger condition first activates. The minimum acceleration of RVTL when the trigger condition activates is shown in Supplementary Fig.3. The result shows that the simple distance threshold cannot determine the "not impeding" since some drivers may still perform significant deceleration even with a rather long distance. Furthermore, the data demonstrates that the distance to RVTL is related to the speed difference. Thus, the relation of the distance to the speed difference is selected to determine the compliance threshold.

According to Fig.5g in the main paper, the interfered points are all close to the bottom-left corner and exhibit a negative correlation between distance and relative velocity. Therefore, three common forms of function curves were selected and optimized using a genetic algorithm, the function forms are as follows: 1) linear function: $d_{\text{cl\_min}} = a{\cdot}x{+}b$, 2) power function: $d_{\text{cl\_min}} = a(x+b)^c + d$, 3) exponential function: $d_{\text{cl\_min}} = a \cdot e^{(bx)} + c$.

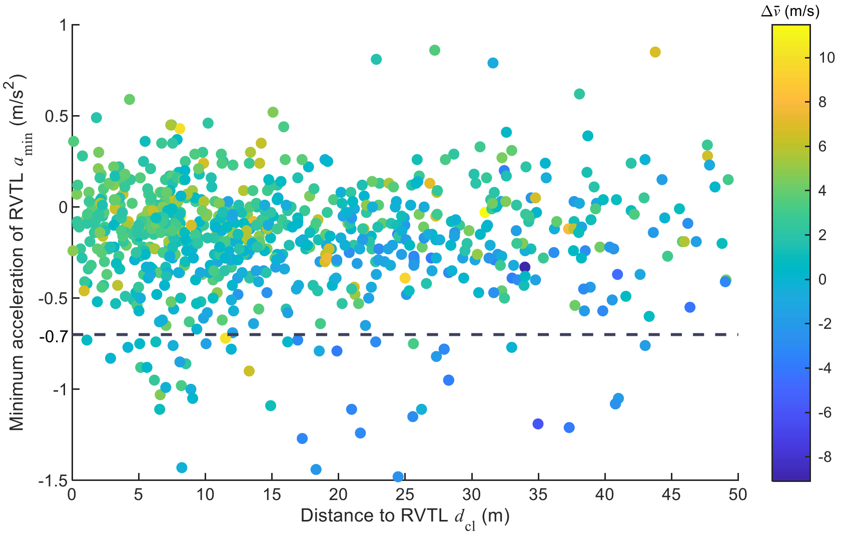

Fig. 3 The minimum acceleration of RVTL activates during the trigger condition. The x-axis represents the longitudinal distance when the trigger condition activates, the y-axis represents the minimum acceleration of RVTL during the trigger condition activates, and the color represents the average speed difference between the two vehicles during the trigger condition activates.

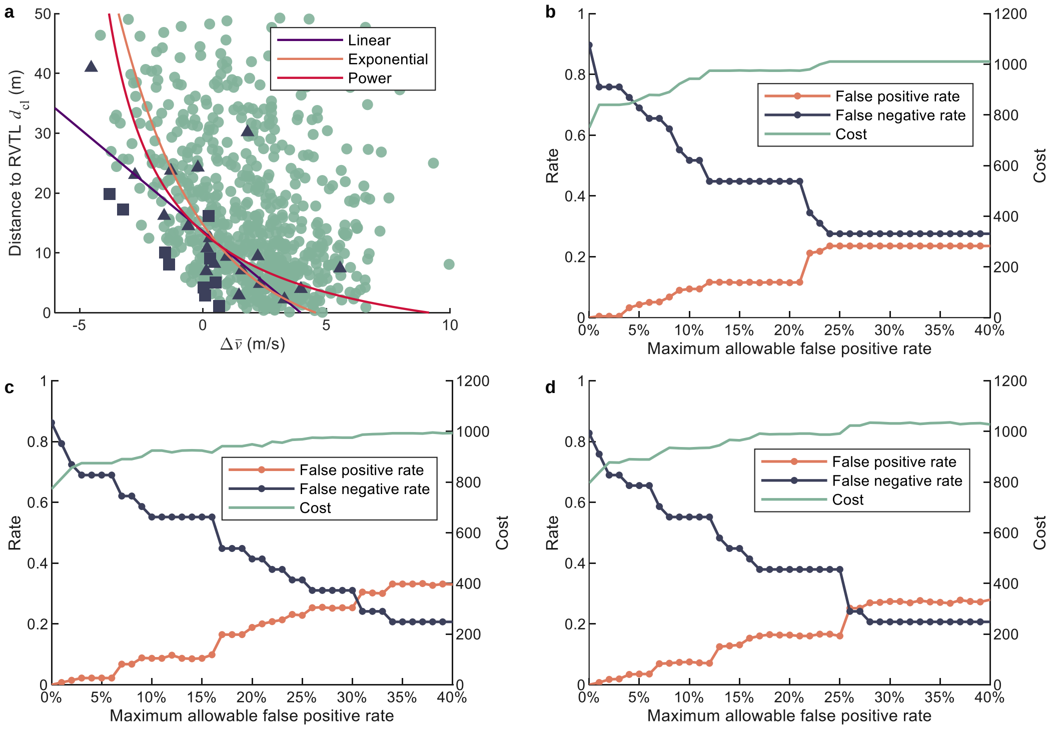

Fig. 4 The optimal results of three function types. a. Optimal threshold lines for the three functions, each ensuring the maximum cost. **b.** Optimization results for linear function at different maximum allowable false positive rate. **c.** Optimization results for power function at different maximum allowable false positive rate. **d.** Optimization results for exponential function at different maximum allowable false positive rate.

The results are shown in Supplementary Fig.4. Supplementary Fig.4a shows the optimal curves for the three optimized functions, each ensuring the maximum cost ($N_{\mathrm{TP}} \cdot Q + N_{\mathrm{TN}}$). Supplementary Fig.4b-d represent the optimization results for linear, power, and exponential functions at different maximum allowable false positive rates. The result shows that the linear function reaches the maximum cost value of 1010.7 when the false-positive rate is 24%, at this time, the numbers of false-positive and false-negative are 160 and 8. The maximum cost of the power function is 995.5 with a 34% false-positive rate and the numbers of false-positive and false-negative are 225 and 6. For exponential function, the maximum cost is 1035.5 with a 28% false-positive rate and the numbers of false-positive and false-negative are 183 and 6. Therefore, the linear function has the lowest false-positive rate at the maximum cost value, and the exponential function can achieve the highest cost value. But when the exponential function reaches the maximum cost value, the false-positive rate is higher than the false-negative rate, which is undesirable for monitoring.

In the data where the false-positive rate is less than the false-negative rate, the linear function has the maximum cost value. After comprehensive consideration, we chose this linear function as the final threshold.

3.3 Some interesting findings

Due to the lack of effective continuous monitoring, most rule violations get penalties only when incidents occur. Consequently, on highways, many drivers tend to prioritize efficiency over compliance. For instance, highway regulations stipulate a minimum 50 m following distance between vehicles, but the dataset reveals that most drivers fail to maintain this distance. Moreover, since rear-end collisions typically hold the rear vehicle responsible, a large majority of drivers pay less attention to maintaining distance from the RVTL when making lane-change. Among vehicles engaged in lane-change violations, most drivers are unable to maintain the safe distance from RVTL, even exhibiting highly aggressive cut-in behavior with a distance as little as 0.1 times the theoretical RSS distance.

However, as indicated in Fig.5c in the main paper, even exceptionally aggressive drivers will maintain a certain distance from RVTL during lane-changes, based on relative velocity. Below this threshold, all vehicles will be engaged in other driving behaviors. Furthermore, since most vehicles rarely perform significant deceleration during high-speed driving, drivers tend to reduce the distance to the leading vehicle. This might explain why some aggressive drivers only make rapid lane changes when their time gap to the leading vehicle falls below 2 seconds. This is also the reason why most RVTLs do not exhibit noticeable avoidance behavior, even when confronted with dangerously close cut-in.

As shown in Table 1 in the main paper, among all monitoring instances regarding to speed violation, because of the congestion on the road, about 95.06% of the vehicles cannot satisfy the minimum speed requirement while no vehicle is over speed. The same reasons accounted for 84.46% of the monitored vehicles failing to meet the minimum following distance requirements. Such violations indicate that under certain specific working conditions, vehicles will find it challenging to adhere to traffic laws defined for typical situations. Therefore, leveraging the superior data processing capabilities of self-driving vehicles, the subsequent development of more refined traffic laws tailored for self-driving might be a solution. This approach aims to ensure that vehicles have adequate guidelines for driving without violations even in uncommon working conditions.

At the intersections, as seen in Fig.6a in the main paper, it is evident that in left-turn vehicle passes first cases, left-turning vehicles arrive at the intersection mostly 2 seconds ahead of straight-moving vehicles. Instances of left-turning vehicles rush typically occur when left-turning vehicles are around 4-8 s away from the intersection point. Conversely, left-turning vehicles yielding the straight-moving vehicles are commonly observed when they are 2-5 seconds away from the intersection points. Examining the points where the curves intersect the axes, vehicles generally pass the intersection points with a minimum time gap of around 2 s.

Table 3 Traffic light violations in different cities

City	Tianjin		Changchun		Chongqing		Xi'an	
Number of total monitoring instances	3433		4775		1379		3407	
Number and percentage of on stop line at the red light	202	5.88%	116	2.43%	62	4.5%	91	2.67%
Number and percentage of on stop line at the yellow light	13	0.38%	16	0.34%	7	0.51%	18	0.53%
Number and percentage of running the red light	42	1.22%	423	8.86%	10	0.73%	70	2.05%
Number and percentage of running the yellow light	111	3.23%	63	1.32%	30	2.18%	14	0.41%

Supplementary Table 3 shows a more detailed result about the violations of traffic light. It can be observed that there are more instances of red light violations than yellow-light violations. This might be due to the very short duration of yellow lights (approximately 3-5 s). If we consider violations on a time-based scale, there will be more vehicles running yellow lights per second. This is probably because the penalties for running red lights are more severe, and in some cities, running yellow lights goes unpunished. As a result, there is a noticeable regional disparity, as seen in Tianjin, where there are 111 instances of running yellow lights, roughly 2.6 times the number of red light violations. This clearly indicates a lack of penalties for running yellow lights in Tianjin, whereas in Xi'an, there are only 14 instances of running yellow lights, approximately 0.2 times the number of red light violations, indicating stricter enforcement in Xi'an. In the case of the intersection in Changchun, with only 7 data segments, a total of 423 vehicles were found to run red lights, comprising 77.6% of all red light violations among the cities analyzed. Therefore, even though national regulations have been issued, there may be slight variations in the specific enforcement of these regulations in different regions. This leads to variations in driving behaviors among drivers in different areas, and it is a challenge that might need to be addressed in future compliance monitoring.
