# Supplementary material for: Online legal driving behavior monitoring for self-driving vehicles
Source: Nat Commun. 2024 Jan 9;15:408. doi: 10.1038/s41467-024-44694-5 (PMC10776857; doi:10.1038/s41467-024-44694-5)
Supplement: Supplementary file 3 — Description of Additional Supplementary Files [file 41467_2024_44694_MOESM3_ESM.pdf]

Description of Additional Supplementary Files

File Name: Supplementary Movie 1
Description: Scenario in which the ego vehicle first follows the target vehicle and then performs an aggressive overtaking maneauver. Drone perspective.

File Name: Supplementary Movie 2
Description: Scenario in which the ego vehicle first follows the target vehicle and then performs an aggressive overtaking maneauver. Ego vehicle perspective.

File Name: Supplementary Movie 3
Description: Scenario in which the ego vehicle initially maintains its lane normally and then changes to drive on the line for a long time. Drone perspective.

File Name: Supplementary Movie 4
Description: Scenario in which the ego vehicle initially maintains its lane normally and then changes to drive on the line for a long time. Ego vehicle perspective.

File Name: Supplementary Code 1
Description: AD4CHE analysis, SIND analysis, Online monitor program. AD4CHE analysis and SIND analysis include the code and output data for dataset analysis. Online monitor program is a MATLAB version of the monitoring of articles related highway.
